# Antinociceptive effects of dehydrocorydaline in mouse models of inflammatory pain involve the opioid receptor and inflammatory cytokines

**DOI:** 10.1038/srep27129

**Published:** 2016-06-07

**Authors:** Zhi-Yu Yin, Lu Li, Shuai-Shuai Chu, Qing Sun, Zheng-Liang Ma, Xiao-Ping Gu

**Affiliations:** 1 Department of Anesthesiology, Affiliated Drum Tower Hospital of Nanjing University Medical School, No.321, Zhongshan Road, Nanjing City, 210008, Jiangsu Province, China

## Abstract

Dehydrocorydaline (DHC) is an alkaloidal component isolated from *Rhizoma corydalis*. Previous studies have shown that DHC has anti-inflammatory and anti-tumor effects and that it can protect the cardiovascular system. However, there are few studies of the antinociceptive effects of DHC *in vivo*. This study explored the antinociceptive effects and possible mechanisms of DHC in mice using two inflammatory pain models: the acetic acid-induced writhing test and the formalin paw test. The intraperitoneal administration of DHC (3.6, 6 or 10 mg/kg) showed a dose-dependent antinociceptive effect in the acetic acid-induced writhing test and significantly attenuated the formalin-induced pain responses in mice. The antinociceptive effects of DHC were not associated with changes in the locomotor activity or motor responses of animals, and no obvious acute or chronic toxic effects were observed in the mice. Furthermore, the use of naloxone confirmed the involvement of the opioid receptor in the central antinociceptive effects of DHC. DHC reduced formalin-induced paw edema, which indicated that DHC may produce an anti-inflammatory effect in the periphery. In the formalin test, DHC decreased the expression of caspase 6 (CASP6), TNF-α, IL-1β and IL-6 proteins in the spinal cord. These findings confirm that DHC has antinociceptive effects in mice.

Pain is a serious problem globally. It has been estimated that 1 in 5 adults suffers from pain and that another 1 in 10 adults is diagnosed with chronic pain each year^1^. Pain not only reduces the quality of life but also imposes high health costs and economic losses to society. Unfortunately, the available analgesic drugs exert a wide range of side effects, and most are either too potent or too weak^2^. Therefore, the search for new analgesic compounds to serve as therapeutic alternatives is important. For thousands of years, various extracts of natural products, most of which are derived from plants, have been used as analgesics. In most cases, a single natural product contains a complex list of compounds, some of which have biological activities. Thus, studying plant extracts that traditionally have been used as painkillers could be seen as a suitable alternative strategy in the search for new analgesic drugs.

Dehydrocorydaline (DHC) (Fig. 1) is an alkaloidal component isolated from *Rhizoma corydalis* (the dried tuber of *Corydalis yanhusuo W.T. Wang*)^3^. In the traditional Chinese medical system, *Rhizoma corydalis* has been used as an analgesic to treat, for example, spastic pain, abdominal pain and pain due to injury for thousands of years^4^, and no toxicity has been reported. Many previous studies, including animal experiments and clinical trials, have shown that DHC exerts protective effects on the cardiovascular system^5^. In addition, the anti-allergic^6^ and anti-tumor^7^ effects of DHC have been confirmed. Recently, Kazuhiro Ishiguro’s study showed that DHC decreases the production of tumor necrosis factor (TNF-α) and interleukin (IL)-1β in RAW 264.7 cells and inhibits the production of IL-6 in fibroblast-like synoviocytes^8^. These results demonstrate that DHC possesses anti-inflammatory effect. It is commonly believed that proinflammatory cytokines such as TNF-α, IL-1β and IL-6 are involved in the pain process and that their peripheral and central levels are up-regulated in many pain models^9^. On this basis, we hypothesized that DHC, one of the main constituents of *Rhizoma corydalis*, might be a contributing factor in the medicinal uses of *Rhizoma corydalis* for relieving pain. However, few reports are available on the antinociceptive effect of DHC. The acetic acid-induced writhing test and the formalin-induced pain test have been the most frequently used standard screening methods in the discovery of new analgesics^10^,^11^. The present experiments were designed to investigate the antinociceptive effect of DHC and to explore its possible underlying mechanism using these two tests of pain in mice.

## Results

### Effects of DHC on the acetic acid-induced writhing test in mice

#### The onset time of writhing in mice

**Figure 2:**
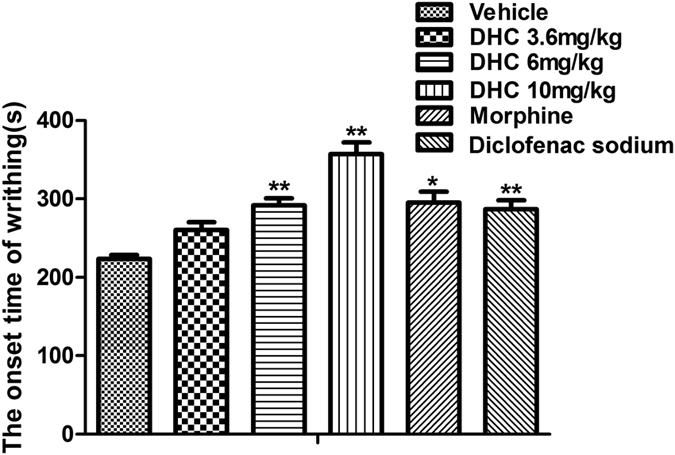
DHC extended the onset time of the acetic acid-induced writhing responses in mice. *P < 0.05, **P < 0.01 versus the vehicle group. n = 8–12.

As shown in Fig. 2, an intraperitoneal injection of DHC (3.6, 6 or 10 mg/kg) delayed the onset time of writhing in mice in a dose-dependent manner. These data also demonstrated that there were no significant differences among the onset times of writhing after giving DHC (6 and 10 mg/kg), morphine (1 mg/kg), or diclofenac sodium (10 mg/kg).

#### Numbers of writhing episodes in mice

**Figure 3:**
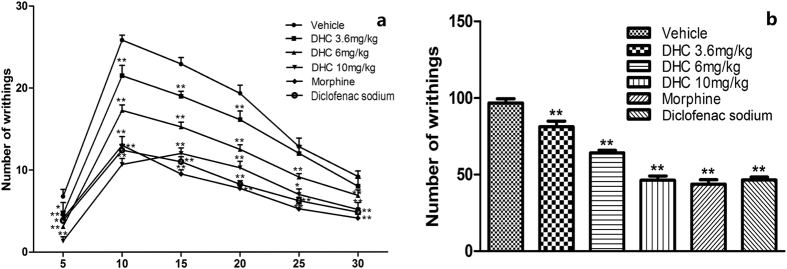
(**a**) The interaction plot of time and group in the writhing test, *P < 0.05, **P < 0.01, versus the vehicle group in the same time intervals. n = 8–12. (**b**) Total number of writhing episodes in 30 min after acetic acid injection, *P < 0.05, **P < 0.01 versus the vehicle group. n = 8–12.

Repeated measures ANOVA tests indicated that there was a time effect (F = 311.609, *P* < 0.01) and a group effect (F = 74.191, *P* < 0.01) as well as a time × group interaction effect (F = 9.188, *P* < 0.01). The time and group interaction plot is shown in Fig. 3. Overall, DHC (3.6, 6 and 10 mg/kg, i.p.) attenuated the numbers of acetic acid-induced writhing episodes in a dose-dependent manner (*P* < 0.01, Fig. 3). Treatment with DHC at doses of 3.6, 6 or 10 mg/kg led to 16.5% (*P* < 0.01), 34.0% (*P* < 0.01) and 52.6% (*P* < 0.01) decreases in the writhing responses, respectively, compared with the control group. Similar to the effect of 10 mg/kg DHC, morphine (1 mg/kg) and diclofenac sodium (10 mg/kg) produced 54.6% and 52.0% decreases in the number of writhing episodes, respectively.

#### Effects of DHC on formalin paw test in mice

**Figure 4:**
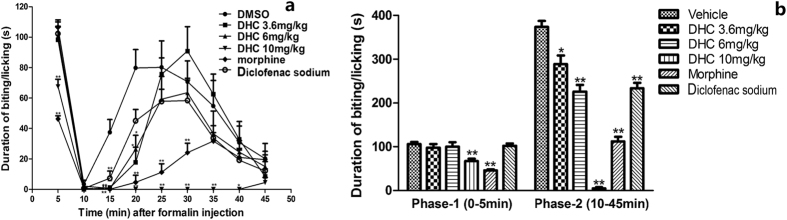
(**a**) The interaction plot of time and group in the formalin paw test. *P < 0.05, **P < 0.01 versus the vehicle group in the same time intervals. n = 8~12. (**b**) Duration of biting/licking after the formalin injection in Phase-1 and Phase-2. *P < 0.05, **P < 0.01 versus the vehicle group. n = 8–12.

To confirm and extend our understanding of the antinociceptive efficacy of DHC, we performed the formalin paw test. The formalin paw test produces a distinct biphasic response^12^. Phase-1 (the early phase) occurs within the first 5 minutes and Phase-2 (the late phase) occurs from 10 minutes to 45 minutes after formalin injection. Repeated measures ANOVA tests indicated that there was a time effect (F = 57.543, *P* < 0.01) and a group effect (F = 109.630, *P* < 0.01) as well as a time × group interaction effect (F = 3.437, *P* < 0.01). The time and group interaction plot is shown in Fig. 4a. Figure 4b shows that DHC caused a significant dose-dependent reduction (*P* < 0.01) in the amount of time the mice spent on licking/biting in the Phase-2 and that the effect of the high dose of DHC was comparable to that of morphine and diclofenac sodium. In Phase-1, consistent the results of others, our test demonstrated that morphine significantly reduced the pain responses. Interestingly, only the highest dose of DHC (10 mg/kg) showed an antinociceptive effect in Phase-1, and this effect was weaker than that of morphine.

#### Effects of DHC on locomotor activity and motor responses

**Figure 5:**
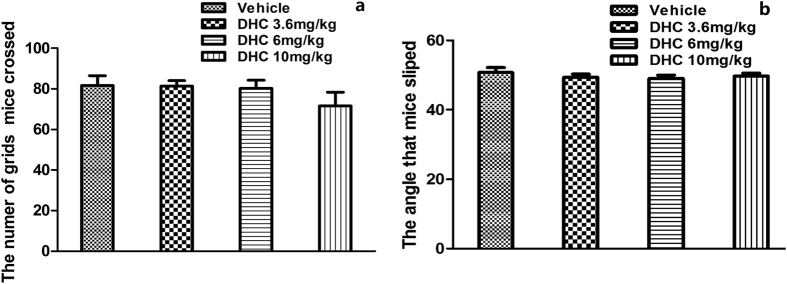
DHC had no influence on the locomotor activity and motor responses. *P < 0.05, **P < 0.01 versus the vehicle group. n = 8–12.

To rule out the possibility that the DHC-induced antinociceptive effects were the secondary to sedative or muscle-relaxant effects, we tested the effects of DHC on locomotor activity and motor responses. As shown in Fig. 5, the dose of DHC capable of producing intense antinociceptive effects has no significant (*P* > 0.05) influence on locomotor activity or motor responses compared with the control group.

#### Safety evaluation: Acute and chronic toxicity of DHC

The administration of DHC (i.p.) did not cause any aberrant behaviors or abnormalities in the diet, body weight, hair, feces, activity or gross anatomy in the mice. No significant effects were observed during the first 3 days or the following 14 days. These results indicate that DHC did not cause any acute or chronic toxicity.

#### Involvement of the opioid system in the antinociceptive effect of DHC

**Figure 6:**
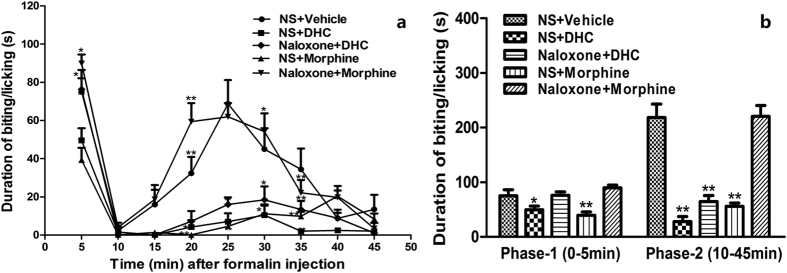
(**a**) The interaction plot of time and group in the formalin paw test to evaluate the involvement of opioid receptors, *P < 0.05, **P < 0.01 versus the control group in the same time intervals. n = 8. (**b**) Duration of biting/licking after the formalin injection in Phase-1 (0–5 min) and Phase-2 (10–45 min). *P < 0.05, **P < 0.01 versus the control group. n = 8.

Figure 6 shows that compared with the control group, DHC (10 mg/kg) demonstrated significant antinociceptive activity (*P* < 0.05) and that the effect was comparable to that of the reference drug morphine (10 mg/kg) in both Phase-1 and Phase-2. Repeated measures ANOVA tests indicated that there was a time effect (F = 38.696, *P* < 0.01) and a group effect (F = 14.827, *P* < 0.01) as well as a time × group interaction effect (F = 2.790, *P* < 0.01). The antinociceptive activities of morphine in the two phases were completely antagonized by naloxone (*P* < 0.05). Also, naloxone significantly decreased the antinociceptive effects produced by 10 mg/kg DHC (*P* < 0.05). It is worth noting that in Phase-1, naloxone completely prevented the antinociceptive effects of DHC whereas in Phase-2, naloxone only partially prevented the effects.

#### Effects of DHC on paw edema in mice

**Figure 7:**
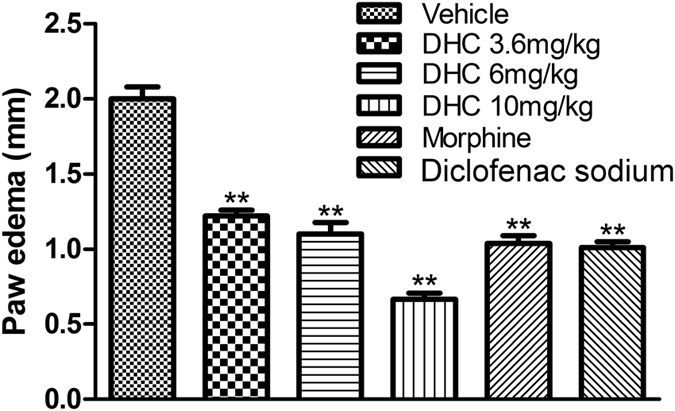
DHC reduced the paw edema induced by formalin in mice. *P < 0.05, **P < 0.01 versus the vehicle group. n = 8–12.

Figure 7 shows that DHC caused a significant (*P* < 0.01) dose-dependent reduction in the formalin-induced increase in the paw thickness in the mice. In the control group, the paw thickness increased to 2.00 ± 0.08 mm whereas in the 10 mg/kg DHC group the thickness was 0.67 ± 0.42 mm, thus producing a 66.8% (*P* < 0.01) reduction in edema formation. Furthermore, the effect of the 10 mg/kg dose of DHC was greater than that of the reference drug diclofenac sodium (20 mg/kg). The results confirmed that DHC demonstrates an anti-inflammatory effect in the periphery.

#### Involvement of the CASP6/TNF-α pathway, IL-1β and IL-6 in the spinal cord

**Figure 8:**
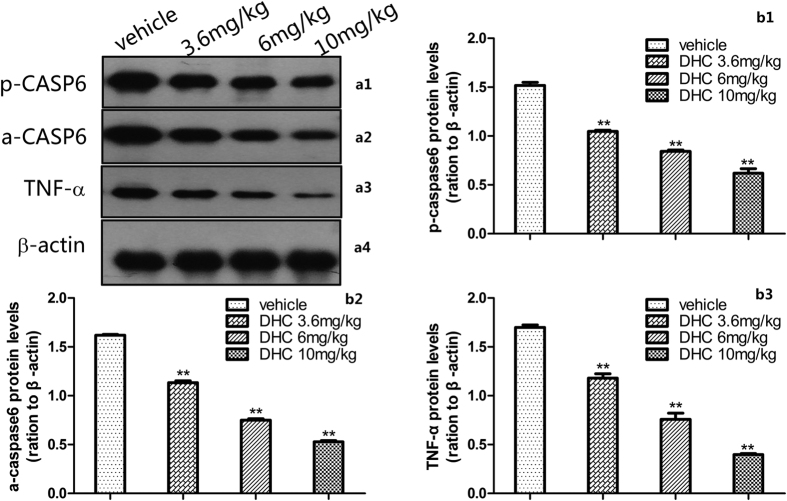
p-CASP6, a-CASP6 and TNF-α expression 60 min after the formalin injection. *P < 0.05 and **P < 0.01 versus the vehicle group. n = 3. (a1–a3) Representative Western blots of p-CASP6, a-CASP6 and TNF-α in the spinal cord. (b1–b3) Quantification of the levels of p-CASP6, a-CASP6 and TNF-α expression. The blots were run under the same experimental conditions.

**Figure 9:**
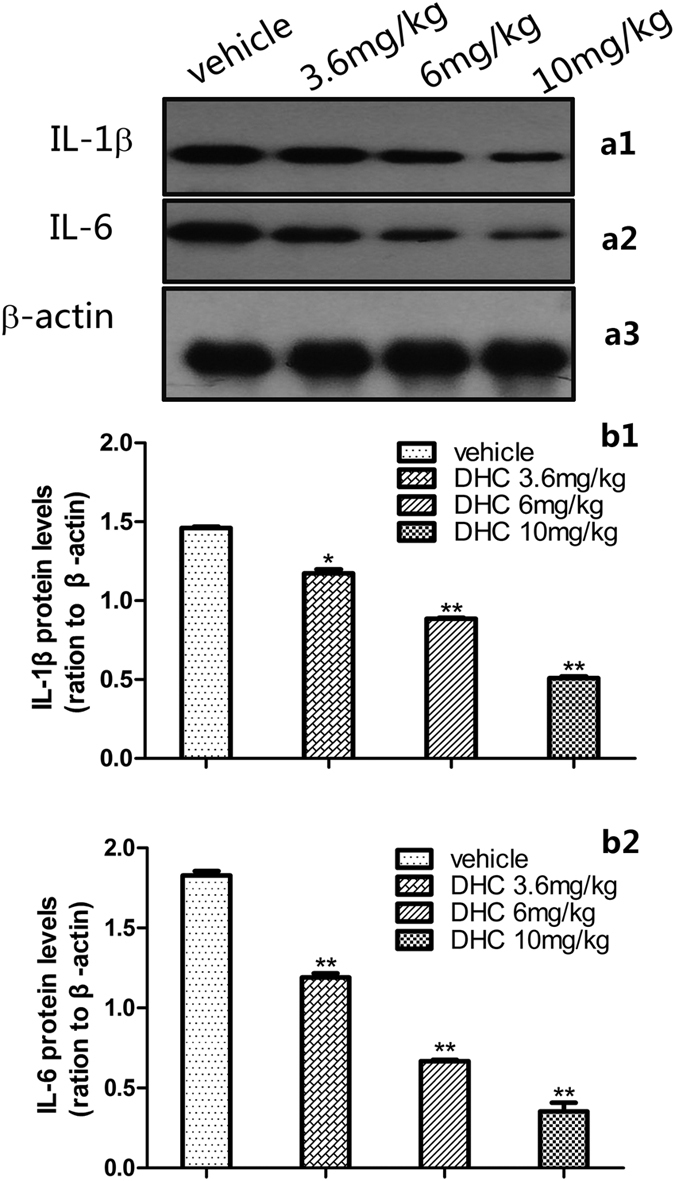
IL-1β and IL-6 expression 60 min after the formalin injection. (a1–a3) Representative Western blots of IL-1β and IL-6 in the spinal cord. (b1,b2) Quantification of the levels of IL-1β and IL-6 expression. The blots were run under the same experimental conditions.

In the formalin test, DHC caused a significant dose-dependent (*P* < 0.05) down-regulation of the inflammatory cytokines p-CASP6, a-CASP6, TNF-α, IL-1β and IL-6 proteins in the spinal cord measured 60 min after the injection. Specifically, compared with the control group, 10 mg/kg DHC decreased the expression of p-CASP6, a-CASP6, TNF-α, IL-1β and IL-6 by 59%, 67%, 77%, 65% and 81%, respectively (Figs 8 and 9).

## Discussion

The acetic acid-induced writhing test is a widely used classic non-selective animal model of pain. Acetic acid can produce peritoneal inflammation. The injection of acetic acid directly activates the visceral and somatic nociceptors that innervate the peritoneum and then induces inflammation not only in subdiaphragmatic visceral organs but also in the subcutaneous muscle walls^13^. Ronaldo A *et al.* suggested that the nociceptive activity of acetic acid in the writhing model may be due to the release of TNF-α, IL-1β and IL-8 by resident peritoneal macrophages and mast cells^14^. The antinociceptive effects of DHC may result from an inhibition of the production of inflammatory mediators (e.g., TNF-α, IL-1β) released into the peritoneal cavity. Several categories of drugs, including muscle relaxants and adrenergic receptor agonists, can also inhibit writhing^15^. Due to the lack of specificity of this test, positive results in the writhing test require confirmation in other models. In the present study, a formalin test was used for this purpose.

The formalin test is a valid model used in pain and analgesia research and has been reported to produce distinct biphasic nociceptive responses. The first phase (0 to 5 min) is believed to be caused by non-inflammatory pain resulting from direct stimulation of nociceptors. The second phase (10 to 45 min) is thought to be an inflammation-induced pain that is associated with inflammatory cytokines in the periphery and spinal cord^12^. In general, centrally acting drugs inhibit both phases, whereas peripherally acting drugs inhibit only the second phase. On the basis of the experiments in our study, DHC dose-dependently and significantly suppressed the second phase pain whereas only the 10 mg/kg dose of DHC showed an analgesic effect on the first phase. The reduction of the first phase demonstrates that DHC may possess a central antinociceptive effect. The pronounced effects of DHC in the inflammatory phase (Phase-2) indicate the possibility of substantial involvement of inflammatory mediators in the periphery or spinal cord. The results from the formalin acetic acid-induced writhing tests confirmed the antinociceptive effects of DHC. In fact, DHC (10 mg/kg) was more potent than diclofenac and even more potent than morphine in the formalin test. As a result, we suggest that the effect of DHC had opioid characteristics. The subsequent experiments were carried out to further investigate the possible central or peripheral mechanisms of DHC.

The results showed a rapid onset of the effects of DHC after administration. One possible explanation for this phenomenon may be the solubility of the DHC, which allows it to rapidly reach the analgesic site. Recent studies have confirmed that after an intraperitoneal injection of mice with a prescription consisting of an extract of Yuanhu Zhitong (YZ), a traditional Chinese formulation composed of *Rhizoma corydalis*, DHC can be detected within minutes in plasma and reaches a peak in the brain after 15 min^16^.

The experimental results from the open-field test and inclined plane test confirmed that DHC caused no detectable skeletal muscle relaxation or sedative effects on the central nervous system. Therefore, the behavioral responses that were observed in the writhing test and formalin paw test were not due to motor dysfunction or sedation but reflected true antinociceptive effects. Importantly, no significant changes were observed in mice that had been given DHC (i.p.), which demonstrated the safety of DHC. In fact, DHC manifests a low acute toxicity with an LD_50_ of about 277.5 ± 19.0 mg/kg body weight in mice following oral administration and 21.1 ± 1.4 mg/kg for intraperitoneal injection^5^.

Among the neurotransmitter systems involved in pain, the opioid system is one of the most important. Jinsmaa’s study demonstrated that the μ2- and δ-opioid receptors are involved in the spinal mechanism, whereas the μ1/μ2-opioid receptors may mediate mainly supraspinal analgesia^17^ in the formalin paw test. To further address the possible central antinociceptive mechanisms of DHC, we examined the effects of naloxone, a non-selective opioid receptor antagonist, on the effects of DHC. The results of the formalin test indicated that the antinociceptive effects of DHC on the Phase-1 responses were completely prevented by naloxone, whereas the Phase-2 responses were partially prevented by naloxone. In addition to the opioid receptor-mediated effects, other mechanism(s) may also take part in the Phase-2 responses. We hypothesized that DHC may reduce the inflammatory cytokines in the paw and spinal cord. The next experiment was designed to further validate the proposed hypothesis.

Considering the results above, we suggested an anti-inflammatory action of the DHC in the periphery. It is well known that the local injection of formalin produces an inflammatory response that results in the paw swelling^18^. The acute inflammation induced by formalin is caused by cell damage that provokes the production of endogenous mediators and subsequently causes the release of a series of inflammatory mediators in the paw^19^. DHC caused a significant dose-dependent reduction in edema comparable to that induced by the standard reference drug diclofenac sodium. These results demonstrate a possible inhibition of inflammatory mediators released in the periphery.

Recently, substantial evidence has shown that the glial cells in the spinal cord create and maintain sensitization to inflammatory pain by releasing potent neuromodulators including pro-inflammatory cytokines (e.g., TNF-α, IL-1β and IL-6)^20^,^21^. Furthermore, the roles of TNF-α, IL-1β and IL-6 in the sensitization to inflammation-associated pain have been well demonstrated. It is noteworthy that Temugin Berta’s latest study found that CASP6 is expressed specifically in C-fiber axonal terminals in the superficial dorsal horn of the spinal cord and that animals exposed to an intra-plantar formalin injection exhibited CASP6 activation in the dorsal horn. The increased concentration of CASP6 can activate microglial TNF-α secretion and regulate synaptic plasticity and inflammatory pain^22^. One major observation made in the current investigation is that the CASP6/TNF-α pathway, particularly the expression of CASP6, is involved in the pain response, because the activation of CASP6 plays a pivotal role in the induction and maintenance of central sensitization. The study by Gruber *et al.* demonstrated that TNF-α is required for the induction of spinal long-term potentiation (LTP) and inflammatory pain^23^. In addition to direct modulation of synaptic transmission, TNF-α further stimulates glial cells to release pro-inflammatory mediators including IL-1β and IL-6 that enhance synaptic transmission and LTP^24^. In the present study, the increased levels of CASP6 (including total CASP6 and active CASP6) and TNF-α in the spinal dorsal horns of the mice in the formalin test were attenuated by the DHC treatment. These results suggest that the antinociceptive effects of DHC may partly act via inhibition of the CASP6/TNF-α pathway.

Finally, we showed that in the formalin test, DHC significantly altered the IL-1β and IL-6 protein levels in the spinal cord. As mentioned above, in the inflammatory pain state, the glial cells are activated and release inflammatory mediators (e.g., IL-1β and IL-6) in the peripheral nervous system (PNS) or central nervous system (CNS), which may play a crucial role in driving, maintaining and aggravating inflammatory pain^25^. We speculate that DHC is an anti-inflammatory pain agent that specifically affects the CASP6/TNF-α pathway and thereby reduces the levels of inflammatory cytokines such as IL-1β and IL-6 in the spinal cord.

In conclusion, DHC exerts antinociceptive effects as confirmed by the acetic acid–induced writhing test and formalin paw test. To our knowledge, this is the first report of the antinociceptive effects of DHC. Opioid receptors and inflammatory cytokines (CASP6/TNF-α, IL-1β and IL-6) in the spinal cord may be involved in the antinociceptive effects of DHC. DHC may be a potentially novel treatment useful for inflammatory pain. Considering that DHC is one of the main constituents of the corydalis tuber, DHC may be a contributing factor in the medicinal uses of *Rhizoma corydalis* in pain treatment. Further studies are needed to substantiate the present data and to explore more precise mechanisms in these important antinociceptive effects of DHC.

## Methods

### Animals

Adult male ICR mice weighing 25–35 g were obtained from the Laboratory Animal Center of Drum Tower Hospital for use in this study. The animals were kept under standard environmental conditions of controlled temperature (22 ± 2 °C) and humidity with a 12 h light/dark cycle. Food and water were supplied ad libitum. All experiments were approved by the Animal Care and Use Committee at the college and were in accordance with the guidelines for the use of laboratory animals. Best efforts were made to reduce the number of animals used and to minimize their suffering.

### Drugs and chemicals

The chemicals were obtained from the following suppliers: DHC, Purity ≥ 98% (product code: VIC449, Vicmed Biotech Co. Ltd., China); diclofenac sodium for injection (product code: 14051901, Bangmin Pharmaceutical Co. Ltd., China); morphine hydrochloride for injection (product code: 140303-2, Northeast Pharmaceutical Daiichi Pharmaceutical Co., Ltd., China); naloxone hydrochloride for injection (product code: 1310141, Beijing Hua Su Pharmaceutical Co., Ltd., China); glacial acetic acid, ACS grade (product code: N985, Amresco, USA); dimethyl sulfoxide (DMSO), ACS/HPLC (product code: 80205, Beijing Superior Chemicals & Instruments Co. Ltd., China); and formaldehyde solution (product code: F1635, Sigma-Aldrich, USA). The DHC was dissolved in DMSO and then diluted with normal saline (NS) prior to administration. Diclofenac sodium and morphine were diluted with NS as positive controls. The vehicle was given to the control group mice. Naloxone was diluted with NS and used as an antagonist. All drugs were administered intraperitoneally (i.p.). The selected concentrations of DHC (3.6, 6 and 10 mg/kg) were based on LD_50_ for DHC described in the literature^5^ and the results of our preliminary experiments.

### Antinociceptive activity tests

#### Acetic acid-induced writhing test

For the writhing test^26^, the mice were first habituated to a plastic observation chamber for 60 min. Then, the mice were given vehicle, DHC (3.6, 6 or 10 mg/kg, i.p.) or morphine (1 mg/kg, i.p.) 15 min before the test. Diclofenac sodium (10 mg/kg, i.p.) was injected 30 min before the test. Subsequently, the mice were treated i.p. with 1% acetic acid (10 ml/kg)^27^. The time of the onset of the writhing was recorded, and the numbers of writhing episodes in each 5 min in the 30 min period beginning at the time of the acetic acid administration were counted. Writhing was marked by contraction of the abdomen and extension of the trunk and hind limbs. The percentage inhibition of writhing was calculated using equation (1).


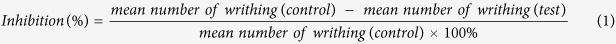


#### Formalin paw test

The formalin paw test was performed according to the methods described by Hunskaar *et al.*^28^. Briefly, the mice were placed individually in glass beakers and were allowed to acclimate for 30 min before the test. The vehicle or DHC (3.6, 6 or 10 mg/kg) were injected (10 ml/kg, i.p.) 15 min prior to the formalin injection. Morphine (10 mg/kg) or diclofenac sodium (20 mg/kg) were injected 15 and 30 min, respectively, prior to the formalin injection as positive controls. Then, 25 μl of a 5% formalin solution was injected into the plantar surface of the right hind paw of each mouse^22^. Immediately after the formalin injection, the mice were placed individually in the beakers, and a mirror was placed under the beaker to allow clear observation of the paws of the animals. The time that the animals spent on biting/licking the injected paw was measured with a stopwatch every 5 min and considered as indication of nociception.

#### Evaluation of locomotor activity and motor responses

The possible non-specific sedative or muscle-relaxant effects of DHC were evaluated using the open-field test^29^ and inclined plane test^30^. The open-field test apparatus consisted of a 50 cm × 50 cm × 50 cm box. The floor of the box was divided into 25 equal squares and the number of squares that each mouse crossed with all paws was monitored for 2 min. In addition, to exclude the possibility of muscle-relaxant effects of DHC, we carried out the inclined plane test. Briefly, we placed the animals on a smooth board and then constantly and gradually raised the board until the animal slipped and then recorded the angle. The mice were treated with the vehicle or DHC (3.6, 6 or 10 mg/kg, i.p.) 30 min before the test.

#### Safety evaluation: acute and chronic toxicity

To evaluate safety of DHC, acute and chronic toxicity studies were carried out. Aberrant behaviors and any abnormalities with respect to diet, body weight, hair, feces, activities and gross anatomy of the mice after the intraperitoneal administration of the vehicle or DHC (3.6, 6 or 10 mg/kg) during the first 3 days and the following 14 days was observed.

### Analysis of the possible mechanisms of actions of DHC

#### Involvement of opioid system

The possible involvement of the opioid system in the antinociceptive effects of DHC was examined by injecting naloxone hydrochloride (2 mg/kg, i.p.)^31^, a non-selective opioid receptor antagonist, prior to the administration of either morphine (10 mg/kg) or DHC (10 mg/kg). Then formalin (5%, 25 μl) was injected into the paw 15 min after the administration of morphine or DHC in the established formalin pain model.

#### Paw edema measurement

The peripheral anti-inflammatory activity of DHC was tested by measuring the formalin-induced paw edema according to the method described by Winter *et al.*^32^. Subcutaneous injection of formalin (5%, 25 μl) into the mouse’s right hind paw produced paw edema at the site of injection. The paw edema was assessed by measuring the dorsal-plantar thickness of the foot with a micrometer (Deshen, China, 0.01 mm) before and 60 min after the formalin injection. The mice were intraperitoneally treated with the vehicle, DHC (3.6, 6 or 10 mg/kg, 15 min-pre) or diclofenac sodium (20 mg/kg, 30 min-pre) before the injection of formalin into the paw. The inhibition of edema was calculated accorting to equation (2).


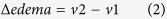


v1: The thickness of paw before injection of formalin.

v2: The thickness of paw after injection of formalin.

#### Involvement of CASPC6/TNF-α pathway, IL-1β and IL-6 in spinal cord

To evaluate the possible involvement of the CASP6/TNF-α pathway, IL-1β and IL-6 in the spinal cord in the antinociceptive actions caused by DHC, the mice were pre-treated (15 min) with the vehicle or DHC (3.6, 6 or 10 mg/kg). The formalin was injected and 60 min later, the lumbosacral enlargement of the spinal cord was rapidly removed for the examination of the levels of pro-CASP6 (the total CASP6), a-CASP6 (the active form of CASP6), TNF-α, IL-1β and IL-6 by western blotting.

#### Western Blotting

The mice were deeply anesthetized with sevoflurane, and the lumbosacral enlargement of the spinal cord was rapidly removed and stored in at −80 °C until further study. The tissue samples were homogenized in lysis buffer with freshly added protease inhibitors. The samples were then incubated on ice for 30 min and centrifuged at 13,000 rpm for 20 min. The supernatants of each sample were collected. The protein concentrations were determined using a BCA Protein Assay Kit, and equal amounts of proteins were separated using SDS-PAGE (8%), following the manufacturer’s instructions. The proteins were subsequently transferred to polyvinylidene fluoride membranes and blocked with 5% skimmed milk in phosphate-buffered saline (PBS) with 0.1% Tween 20 for 2 h at room temperature. After blocking, the membranes were incubated overnight at 4 °C with primary antibodies for CASP6 (1:1,000, Cell Signaling Technology; USA), aCASP6 (active/cleaved, 1:2,000, Imgenex; USA), TNF-α (1:800, Abcam; USA), IL-1β (1:1000, Abcam; USA), IL-6 (1:1000, Abcam; USA), or β-actin (1: 4000; Abcam; USA). The membranes were washed with PBST buffer and incubated with the horseradish peroxidase-coupled secondary antibody (1:5000; Jackson; USA) for 2 h at room temperature. Next, the immune complexes were detected using the ECL system (Santa Cruz Biotechnology, CA, USA). The films were scanned, and the intensities of the selected bands were analyzed using ImageJ software.

### Statistical analysis

The data are presented as the mean ± standard error, and all of the statistical analyses were performed using SPSS 16.0 statistical software. For the numbers of writhing episodes and the licking/biting times, repeated measures ANOVAs and one-way ANOVA followed by the post hoc Bonferroni test were applied. The data for the onset time of writhing, the locomotor activity, the paw edema and the western blots were analyzed using one-way ANOVA followed by the post hoc Bonferroni test. A value of *P* < 0.05 was taken as the level of significance.

### Experimental protocols statement

The study was conducted in accordance with the guidelines, and the Ethics Committee of Affiliated Drum Tower Hospital of Nanjing University Medical School approved the experimental protocols.

## Additional Information

**How to cite this article**: Yin, Z.-Y. *et al.* Antinociceptive effects of dehydrocorydaline in mouse models of inflammatory pain involve the opioid receptor and inflammatory cytokines. *Sci. Rep.* **6**, 27129; doi: 10.1038/srep27129 (2016).

## Figures and Tables

**Figure 1:**
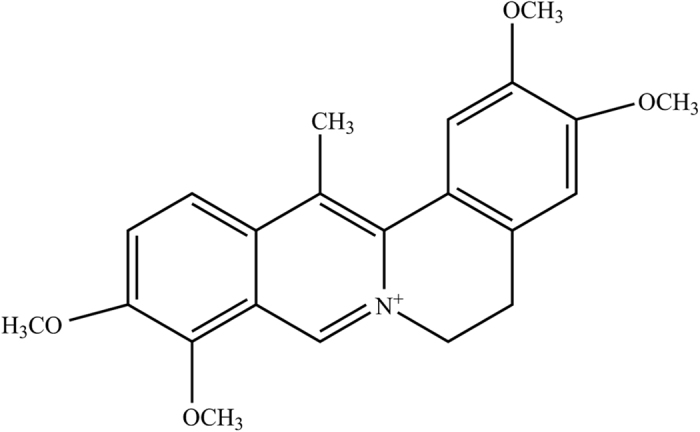
Molecular structure of DHC (C_22_H_24_NO_4_).

## References

[b1] Goldberg D. S. & McGee S. J. Pain as a global public health priority. BMC Public Health. 11, 770 (2011). 21978149 10.1186/1471-2458-11-770 PMC3201926

[b2] Crofford L. J. Adverse effects of chronic opioid therapy for chronic musculoskeletal pain. Nat Rev Rheumatol. 6, 191–197 (2010). 20357788 10.1038/nrrheum.2010.24

[b3] Iranshahy M., Quinn R. J. & Iranshahi M. Biologically active isoquinoline alkaloids with drug-like properties from the genus Corydalis. RSC Adv, 4, 15900–15913 (2014).

[b4] Wang C., Wang S., Fan G. & Zou H. Screening of antinociceptive components in Corydalis yanhusuo W.T. Wang by comprehensive two-dimensional liquid chromatography/tandem mass spectrometry. Anal Bioanal Chem. 396, 1731–1740 (2010). 20101504 10.1007/s00216-009-3409-1

[b5] Jiang X. R. *et al.* Pharmacological actions of dehydrocorydaline on cardiovascular system (author’s transl). Yao Xue Xue Bao. 17, 61–65 (1982). 7090829

[b6] Matsuda H., Tokuoka K., Wu J., Shiomoto H. & Kubo M. Inhibitory effects of dehydrocorydaline isolated from Corydalis Tuber against type I-IV allergic models. Biol Pharm Bull. 20, 431–434 (1997). 9145224 10.1248/bpb.20.431

[b7] Xu Z. *et al.* Dehydrocorydaline inhibits breast cancer cells proliferation by inducing apoptosis in MCF-7 cells. Am J Chin Med. 40, 177–185 (2012). 22298457 10.1142/S0192415X12500140

[b8] Ishiguro K., Ando T., Maeda O., Watanabe O. & Goto H. Dehydrocorydaline inhibits elevated mitochondrial membrane potential in lipopolysaccharide-stimulated macrophages. Int Immunopharmacol. 11, 1362–1367 (2011). 21575743 10.1016/j.intimp.2011.04.022

[b9] Zhang J. M. & An J. Cytokines, inflammation, and pain. Int Anesthesiol Clin. 45, 27–37 (2007). 17426506 10.1097/AIA.0b013e318034194e PMC2785020

[b10] Vincenzi F. *et al.* Antinociceptive effects of the selective CB2 agonist MT178 in inflammatory and chronic rodent pain models. Pain. 154, 864–873 (2013). 23518609 10.1016/j.pain.2013.02.007

[b11] Du J. *et al.* Ligustilide attenuates pain behavior induced by acetic acid or formalin. J Ethnopharmacol. 112, 211–214 (2007). 17350195 10.1016/j.jep.2007.02.007

[b12] Abbott F. V., Franklin K. B. & Westbrook R. F. The formalin test: scoring properties of the first and second phases of the pain response in rats. Pain. 60, 91–102 (1995). 7715946 10.1016/0304-3959(94)00095-V

[b13] Satyanarayana P. S., Jain N. K., Singh A. & Kulkarni S. K. Isobolographic analysis of interaction between cyclooxygenase inhibitors and tramadol in acetic acid-induced writhing in mice. Prog Neuropsychopharmacol Biol Psychiatry. 28, 641–649 (2004). 15276689 10.1016/j.pnpbp.2004.01.015

[b14] Ribeiro R. A. *et al.* Involvement of resident macrophages and mast cells in the writhing nociceptive response induced by zymosan and acetic acid in mice. Eur J Pharmacol. 387, 111–118 (2000). 10633169 10.1016/s0014-2999(99)00790-6

[b15] Le Bars D., Gozariu M. & Cadden S. W. Animal Models of Nociception. Pharmacol Rev. 53, 597–652 (2001). 11734620

[b16] Gao Y., Hu S., Zhang M., Li L. & Lin Y. Simultaneous determination of four alkaloids in mice plasma and brain by LC-MS/MS for pharmacokinetic studies after administration of Corydalis Rhizoma and Yuanhu Zhitong extracts. J Pharm Biomed Anal. 92, 6–12 (2014). 24469095 10.1016/j.jpba.2013.12.037

[b17] Jinsmaa Y. *et al.* Differentiation of opioid receptor preference by [Dmt1]endomorphin-2-mediated antinociception in the mouse. Eur J Pharmacol. 509, 37–42 (2005). 15713427 10.1016/j.ejphar.2004.12.015

[b18] Damas J. & Liegeois J. F. The inflammatory reaction induced by formalin in the rat paw. Naunyn Schmiedebergs Arch Pharmacol. 359, 220–227 (1999). 10208309 10.1007/pl00005345

[b19] Dharmasiri M., Jayakody J., Galhena G., Liyanage S. & Ratnasooriya W. Anti-inflammatory and analgesic activities of mature fresh leaves of Vitex negundo. J Ethnopharmacol. 87, 199–206 (2003). 12860308 10.1016/s0378-8741(03)00159-4

[b20] Taves S., Berta T., Chen G. & Ji R. R. Microglia and spinal cord synaptic plasticity in persistent pain. Neural Plast. 4, 753656 (2013). 24024042 10.1155/2013/753656 PMC3759269

[b21] Schomberg D. & Olson J. K. Immune responses of microglia in the spinal cord: contribution to pain states. Exp Neurol. 234, 262–270 (2012). 22226600 10.1016/j.expneurol.2011.12.021

[b22] Berta T. *et al.* Extracellular caspase-6 drives murine inflammatory pain via microglial TNF-alpha secretion. J Clin Invest. 124, 1173–1186 (2014). 24531553 10.1172/JCI72230 PMC3934175

[b23] Gruber-Schoffnegger D. *et al.* Induction of thermal hyperalgesia and synaptic long-term potentiation in the spinal cord lamina I by TNF-α and IL-1β is mediated by glial cells. J of Neurosci. 33, 6540–6551 (2013). 23575851 10.1523/JNEUROSCI.5087-12.2013 PMC6619063

[b24] Kawasaki Y., Zhang L., Cheng J. K. & Ji R. R. Cytokine mechanisms of central sensitization: distinct and overlapping role of interleukin-1beta, interleukin-6, and tumor necrosis factor-alpha in regulating synaptic and neuronal activity in the superficial spinal cord. J Neurosci. 28, 5189–5194 (2008). 18480275 10.1523/JNEUROSCI.3338-07.2008 PMC2408767

[b25] Wieseler-Frank J., Maier S. & Watkins L. Central proinflammatory cytokines and pain enhancement. Neurosignals. 14, 166–174 (2005). 16215299 10.1159/000087655

[b26] Hokanson G. Acetic acid for analgesic screening. J. Nat. Prod. 41, 497–498 (1978).

[b27] Uddin G. *et al.* Anti-nociceptive, anti-inflammatory and sedative activities of the extracts and chemical constituents of Diospyros lotus L. Phytomedicine. 21, 954–959 (2014). 24703326 10.1016/j.phymed.2014.03.001

[b28] Hunskaar S., Fasmer O. B. & Hole K. Formalin test in mice, a useful technique for evaluating mild analgesics. J Neurosci Methods. 14, 69–76 (1985). 4033190 10.1016/0165-0270(85)90116-5

[b29] Rodrigues A. L. *et al.* Involvement of monoaminergic system in the antidepressant-like effect of the hydroalcoholic extract of Siphocampylus verticillatus. Life Sci. 70, 1347–1358 (2002). 11885577 10.1016/s0024-3205(01)01498-9

[b30] Fehlings M. G. & Tator C. H. The effect of direct current field polarity on recovery after acute experimental spinal cord injury. Brain Res. 579, 32–42 (1992). 1623405 10.1016/0006-8993(92)90738-u

[b31] Brandao M. S. *et al.* Antinociceptive effect of Lecythis pisonis Camb. (Lecythidaceae) in models of acute pain in mice. J Ethnopharmacol. 146, 180–186 (2013). 23276784 10.1016/j.jep.2012.12.028

[b32] Winter C. A., Risley E. A. & Nuss G. W. Carrageenin-induced edema in hind paw of the rat as an assay for antiiflammatory drugs. Proc Soc Exp Biol Med. 111, 544–547 (1962). 14001233 10.3181/00379727-111-27849

